# Gelsolin domains G2G3 recruit other domains and prime the actin filament for severing

**DOI:** 10.1016/j.jbc.2026.113413

**Published:** 2026-08-07

**Authors:** Andrew J. Saks, Malgorzata Boczkowska, Kyle R. Barrie, Robert C. Robinson, Roberto Dominguez

**Affiliations:** 1 Department of Physiology, Perelman School of Medicine, University of Pennsylvania, Philadelphia, Pennsylvania, USA; 2 School of Biomolecular Science and Engineering (BSE), Vidyasirimedhi Institute of Science and Technology (VISTEC), Rayong, Thailand

**Keywords:** actin, gelsolin, filament severing, cryo-electron microscopy (cryo-EM), cytoskeleton dynamics

## Abstract

Gelsolin, a Ca^2+^-dependent actin filament (F-actin) severing protein, plays essential roles in apoptosis, cell motility, and the actin-scavenger system of the bloodstream. It comprises six related domains (G1 to G6) connected by variable linkers (L1 to L5), each contributing distinctly to severing. Individually, neither G1 nor G2G3 severs F-actin, whereas the combined fragment G1–G3 displays potent, Ca^2+^-independent severing activity, largely mediated by G1, which relies on G2G3 for recruitment to F-actin. The C-terminal fragment G4–G6 neither binds nor severs F-actin but, once recruited *via* G2G3, contributes to the severing efficiency of full-length gelsolin. However, it remains unclear whether the other domains rely on G2G3 solely for recruitment or also for conformational changes in F-actin that facilitate binding and severing. Here, through a series of cryo-electron microscopy (cryo-EM) structures, we show that G2G3 binds F-actin in a stepwise manner, inducing conformational changes, including a kink in the filament and rotation of a subunit at the binding site, that prime F-actin for binding by the other gelsolin domains and severing. These changes propagate beyond the G2G3 binding site, revealing how F-actin buffers the effects of kinking. Biochemically, substituting G1 with G4 within G1–G3 yields a construct, G4–G3, with substantial severing activity, albeit Ca^2+^-dependent and lower than that of G1–G3. This result indicates that G4 can substitute for G1 when primed by G2G3, whereas sequence adaptations make G1 uniquely effective at severing. Collectively, these findings define a dual role of G2G3: recruiting other gelsolin domains and priming F-actin for severing.

The actin cytoskeleton drives processes such as cell and organelle motility, endocytosis, and cytokinesis, which depend on the dynamic turnover of actin between its monomeric (G-actin) and filamentous (F-actin) forms. Actin turnover is governed by several families of actin assembly and disassembly proteins that respectively promote the formation of new filament networks and recycle monomers for subsequent rounds of polymerization. Two families of proteins are primarily responsible for filament disassembly: the cofilin family, regulated through phosphorylation and dephosphorylation, and the Ca^2+^-regulated gelsolin family. Although cofilin mediates both filament severing and depolymerization, aided respectively by actin-interacting protein 1 (AIP1) (1, 2) and cyclase-associated protein (CAP) (3, 4), Ca^2+^-bound gelsolin serves a dual role in filament severing and barbed-end capping (5, 6). Gelsolin participates in several cellular processes, ranging from apoptosis (7) to cell motility and shape maintenance (8), to the clearance of actin from the bloodstream as part of the actin-scavenger system (9).

Gelsolin comprises six structurally related domains (G1 to G6) connected by variable linkers (L1 to L5) (6, 10, 11, 12, 13) (Fig. 1*A*). In the absence of Ca^2+^, gelsolin adopts a compact, autoinhibited conformation that masks F-actin–binding sites distributed along the polypeptide (10). Activation occurs progressively with increasing Ca^2+^ concentration, with maximal activity achieved upon Ca^2+^ binding to eight sites, including a conserved type II site within each domain and two type I sites formed at the contact interfaces of G1 and F-actin, and G4 and F-actin (14, 15). Ca^2+^ binding releases autoinhibitory interactions, enabling gelsolin to open and expose its various actin-binding sites (11, 12, 13, 16).

**Figure 1:**
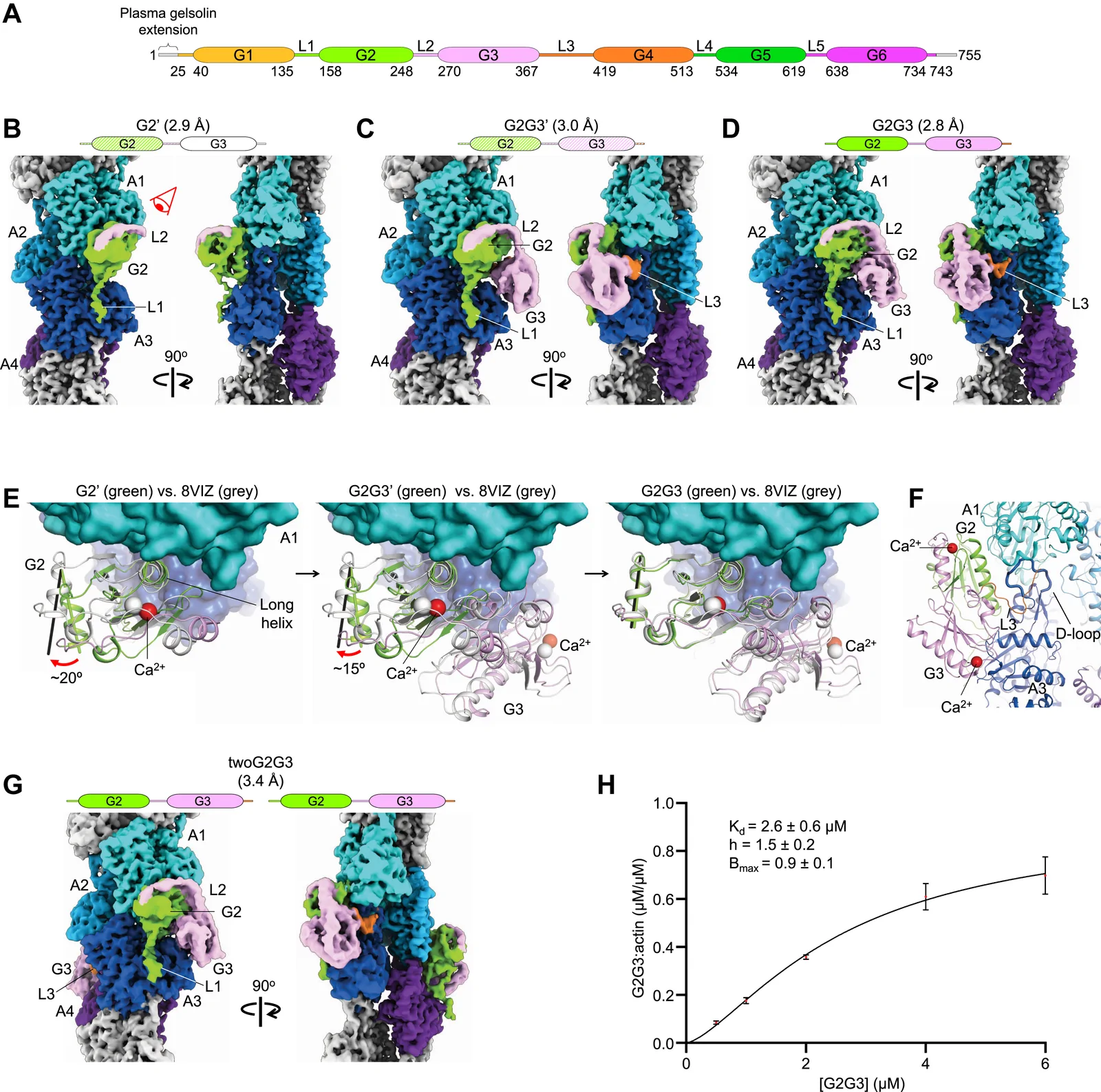
**Stepwise binding of G2G3 to F-actin.***A*, domain diagram of human gelsolin, including the 24–amino acid N-terminal extension of plasma gelsolin. Although cytoplasmic gelsolin lacks this extension, it is numbered by convention starting from M25. (*B*–*D*, and *G*) cryo-EM maps of G2′, G2G3′, G2G3, and twoG2G3 bound to F-actin. Gelsolin domains are colored as in (*A*), and actin subunits are shown in different shades of *blue* or *purple* (interacting with G2G3) or *gray* (noninteracting). Domain diagrams above each map indicate whether domains G2 and G3 are bound (*solid* colors), partially bound (*dashed* colors), or unbound (not colored). *E*, transition of G2 and G3 from unbound to partially bound and fully bound states in the G2′, G2G3′, and G2G3 structures (viewed according to eye icon in part (*B*), with rotations indicated by a *red arrow*) as compared to the barbed end-bound structure of full-length gelsolin (*gray)* (13). (*F*), gelsolin linker L3 interacts with the D-loop of subunit A3. *H*, quantification of the ratio of G2G3 to F-actin (6 μM) in the pellet fraction across a range of G2G3 concentrations, determined from cosedimentation experiments and densitometric analysis of SDS–PAGE (n = 3; Fig. S8). The data were fit in GraphPad Prism version 11.0.0 using nonlinear regression to a specific binding model with a Hill slope, yielding an apparent *K*_*d*_ of 2.6 ± 0.6 μM and a Hill coefficient of 1.5 ± 0.2. F-actin, filamentous actin; cryo-EM, cryo-electron microscopy.

The six gelsolin domains play distinct roles during severing. G1 binds G-actin in a Ca^2+^-independent manner but is largely unable to bind or sever F-actin on its own (17, 18, 19). Severing requires recruitment of G1 to F-actin, which can be minimally supported by linker L1 (19), or even the unrelated actin-binding domain of α-actinin (20). G2G3 binds F-actin but lacks severing activity (17, 18). However, together G1 and G2G3 (N-terminal fragment G1–G3) account for most of gelsolin’s severing activity (21, 22), largely mediated by G1, which depends on G2G3 for recruitment to F-actin (19, 23). In contrast to full-length gelsolin, severing by G1–G3 is Ca^2+^-independent (7, 22, 23, 24), explaining why generation of this fragment during apoptosis *via* caspase-3 cleavage leads to dysregulated F-actin severing, cell rounding, and nuclear fragmentation (7). The C-terminal fragment G4–G6 binds G-actin in a Ca^2+^-dependent manner but neither binds nor severs F-actin (21, 22, 23). However, G4–G6 functions cooperatively with G1–G3 (25), contributing to the greater severing activity of full-length gelsolin relative to any of its fragments (23).

A rather accurate cartoon model for gelsolin-mediated severing was proposed ∼35 years ago (26), in which G1–G3 and G4–G6 form a pincer, engaging the two long-pitch helices on opposite sides of F-actin. Subsequent crystal structures of full-length Ca^2+^-free gelsolin (10), complexes of G-actin with Ca^2+^-bound G1–G3 and G4–G6 (11, 12, 15), and a recent cryo-electron microscopy (cryo-EM) structure of full-length gelsolin at the barbed end of F-actin (13) has refined this model. G2G3 contains the sole F-actin–binding site (17, 18) and is therefore thought to bind first (15), followed by closure of the G1/G4–G6 pincer around F-actin. G1 and G4–G6 are then proposed to competitively displace the DNase I–binding loop (D-loop) from the hydrophobic cleft (H-cleft) of actin subunits in the two long-pitch helices on opposite sides of F-actin. Disruption of this critical intersubunit contact on both sides of the filament, along with thermal fluctuations and a partial flat-to-twisted (F- to G-actin–like) transition in the G1-bound actin subunit, is thought to trigger severing (13, 27). Gelsolin remains bound after severing, capping the newly formed barbed end (13).

Although there is agreement regarding this general model, the specific sequence of steps that leads to severing remains unclear, in particular whether G2G3 functions solely as the initial anchor or also readies F-actin for severing by the other gelsolin domains. Here, we present four cryo-EM structures that reveal how G2G3 binds F-actin in a stepwise manner, culminating in conformational changes that prime the filament for binding and severing by G1 and G4–G6. Consistent with G2G3’s priming role, replacing G5G6 with G2G3 within G4–G6, which otherwise lacks severing activity, yields a chimeric construct (G4–G3) with substantial severing activity, although lower than that of G1–G3. Together, these results establish a dual role for G2G3 in recruiting other gelsolin domains and priming F-actin for severing, while also highlighting the unique role of G1 during severing.

## Results and discussion

### G2G3 binds F-actin in a stepwise manner

In the barbed end-bound structure of full-length gelsolin (13), domains G1–G3 interact with two actin subunits along one of the long-pitch helices, whereas G4–G6 binds a third subunit on the opposite helix (Fig. S1). On the G1–G3 side, G1 occupies the barbed end of the terminal actin subunit, L1 extends toward the pointed end of this subunit, and G2G3 interacts at the interface with the subunit above (toward the pointed end). Compared to the free barbed end (28), the G1-bound terminal subunit is rotated by ∼6°, laterally translated by ∼5.5 Å, and its outer domain (subdomains 1 and 2; residues D1-A144 and E334-F375) is displaced by up to 4.5 Å relative to the inner domain (subdomains 3 and 4; residues S145-P333). We asked whether these changes are driven by the interactions with G1, G2G3, or both. To address this question, and specifically to define the role of G2G3 in severing, we determined cryo-EM structures of G2G3 bound to F-actin (Experimental procedures, Fig. S2, Table 1, Movie S1). The G2G3 construct used, corresponding to human gelsolin amino acids S147–S384, comprises half of linkers L1 and L3 on either side of G2G3, which contribute to F-actin binding (13).

**Table 1:** Cryo-EM data collection, refinement, and model validation statistics

Cryo-EM data and processing	G2′	G2G3′	G2G3	twoG2G3
Magnification	81,000	81,000	81,000	81,000
Voltage, keV	300	300	300	300
Total electron exposure, e^−^/Å^2^	NCEF Krios: 50 UPenn Krios: 44	NCEF Krios: 50 UPenn Krios: 44	NCEF Krios: 50 UPenn Krios: 44	NCEF Krios: 50 UPenn Krios: 44
Defocus range, μm	−0.5 to −2.5	−0.5 to −2.5	−0.5 to −2.5	−0.5 to −2.5
Pixel size, Å	0.54	0.54	0.54	0.54
Symmetry	C1	C1	C1	C1
Final no. particles	328,926	183,304	698,879	39,272
Map resolution, Å FSC threshold 0.143 (0.5)	2.86 (3.19)	3.03 (3.40)	2.76 (3.08)	3.44 (3.86)
Resolution range, Å	2.497–42.336	2.727–43.577	2.416–38.732	2.335–56.043
Model refinement				
Resolution refined structure, Å (FSC threshold 0.5)	3.08	3.25	2.99	3.76
Map sharpening B-factor, Å^2^	105.7	105.0	110.4	76.8
Model composition				
No. atoms (nonhydrogen)	24,389	25,236	25,256	27,027
No. residues	3091	3199	3202	3428
No. ligands	MG: 8,CA: 1, ADP: 8	MG: 8, CA: 2 ADP: 8	MG: 8, CA: 2 ADP: 8	MG: 8, CA: 4 ADP: 8
Correlation model versus data				
CC (mask/box/peaks/volume)	0.83/0.81/0.65/0.83	0.85/0.82/0.66/0.84	0.85/0.83/0.70/0.85	0.81/0.81/0.62/0.81
R.M.S. deviations				
Bond lengths, Å (# > 4σ)	0.008 (0)	0.008 (0)	0.005 (0)	0.003 (0)
Bond angles, ° (#> 4σ)	0.718 (0)	0.764 (0)	0.672 (0)	0.595 (0)
Model validation				
MolProbity score	1.47	1.56	1.44	1.39
Clashscore	3.18	3.27	2.95	3.22
Rotamer outliers, %	2.95	3.00	2.59	2.28
Ramachandran plot				
Favored, %	98.16	97.62	97.82	98.20
Allowed, %	1.84	2.38	2.18	1.71
Disallowed, %	0.00	0.00	0.00	0.00
ADP (B-factors)				
Protein, Å^2^ (min/max/mean)	87.2/299.1/148.0	84.8/303.4/149.9	64.0/266.9/122.8	87.8/339.5/172.9
Ligands, Å^2^ (min/max/mean)	100.8/220.6/135.7	98.5/260.0/134.1	72.4/179.7/107.9	110.6/270.0/154.7
Accession codes				
EMDB	EMD-76298	EMD-76299	EMD-76300	EMD-76301
PDB	12BW	12BX	12BY	12BZ

PDB, Protein Data Bank; EMDB, Electron Microscopy Data Bank; FSC, Fourier Shell Correlation; cryo-EM, cryo-electron microscopy.

The cryo-EM maps were obtained by single-particle analysis rather than helical reconstruction, as G2G3 bound F-actin only sparsely despite being present at a 1:1 ratio with actin (10 μM each). Four structures were obtained: three at 2.8 to 3.0 Å resolution, representing consecutive steps in G2G3 binding to one side of F-actin (Fig. 1, *B*–*D*, Table 1, Figs. S3–S5, Movie S1), and one at 3.4 Å resolution showing two G2G3 molecules bound on opposite sides of F-actin (Fig. 1*G*, Table 1, Fig. S6). The local resolution decreases radially from the center of F-actin (Figs. S3–S6), such that the gelsolin domains are generally less well-defined than indicated by these overall resolution values. Since the cryo-EM sample contained both Ca^2+^ (which binds G2G3) and Mg^2+^ (required for actin polymerization), we also determined the structure of undecorated F-actin present in the sample using helical reconstruction at 2.3 Å resolution to establish the identity of the bound cation. This structure was compared with previously reported 2.2-Å structures of Mg^2+^-ADP and Ca^2+^-ADP F-actin (29). The presence of Mg^2+^ or Ca^2+^ leads to subtle structural differences that contribute to the faster polymerization of Mg^2+^-ATP-actin relative to Ca^2+^-ATP-actin, including differences in the conformation of the nucleotide, the position and coordination of the cation, and the S14 loop (D11–L18), which faces the nucleotide phosphates. The conformation of these structural elements is clearly consistent with the presence of Mg^2+^-ADP in the nucleotide cleft in our structures (Fig. S7).

In the G2G3-bound structures, each G2G3 molecule contacts two actin subunits along the long-pitch helix, designated A1 to A4 from the pointed to the barbed end along the short-pitch helix, such that one G2G3 interacts with A1 and A3 and the other with A2 and A4. To assess the consequences of G2G3 binding, we compared the G2G3-bound structures with both our recent 2.06-Å resolution structure of F-actin (Protein Data Bank [PDB]: 9ZZI) (30) and the structure of full-length gelsolin at the barbed end (13), representing the initial and final states during severing, respectively.

The binding of G2G3 to one side of F-actin occurs through a series of three distinct structural states: G2′, G2G3′, and G2G3 (Fig. 1*E*). These states represent a progression from partially (or transiently) bound to fully bound G2G3. In the G2′ structure, only L1 and G2 are observed (Fig. 1*B*), but their positions differ substantially from those in both the fully bound G2G3 structure and full-length gelsolin at the barbed end. Specifically, the long helix of G2 (R207-N223), which directly faces the critical intersubunit contact formed by the H-cleft of A1 and the D-loop of A3, occupies a position similar to that in the fully bound G2G3 structure. However, G2 is rotated by ∼20° around this helix, causing displacements of distal residues of up to ∼7 Å (Fig. 1*E*, left).

In the G2G3′ structure, G3 and L3 are also observed (Fig. 1*C*). The long helix of G2 continues to superimpose well with that in the G2G3 structure, but G2 and G3 remain rotated as a rigid body by ∼15° around this helix, resulting in displacements of distal residues of >5 Å (Fig. 1*E*, middle). In this state, G3 does not significantly contact F-actin. In the fully bound G2G3 structure, the two gelsolin domains and adjacent linkers adopt a conformation similar to that of full-length gelsolin at the barbed end (Fig. 1*D*). The rotation of G2G3 has almost completely disappeared, although some regions, especially G3, are laterally displaced by up to ∼5 Å (Fig. 1*E*, right). In both the G2G3 and barbed end structures, G3 contacts subdomain 1 of actin subunit A3, whereas L3 interacts with the D-loop of this subunit (Fig. 1*F*). As a result, the movements of G3 in these structures are coupled with changes in A3.

In the twoG2G3 structure, a second G2G3 molecule is bound on the opposite side of F-actin from the first and interacts in the same manner with actin subunits A2 and A4 (Fig. 1*G*). Although this state is markedly underrepresented (5.62% of particles, or 39,272 versus 698,879 for one-side binding), the presence of two G2G3 molecules positioned directly across the filament from one another suggests a degree of cooperative binding. To evaluate this possibility, we performed a cosedimentation assay with F-actin (Figs. 1*H* and S8). G2G3 binds F-actin with weak affinity, with an apparent dissociation constant (K_d_) of 2.6 ± 0.6 μM and a Hill coefficient of 1.5 ± 0.2, indicating modest positive cooperativity. Such cooperativity may, at least in part, explain how the N-terminal proteolytic fragment G1–G3 efficiently severs filaments during apoptosis (7) and, in the context of full-length gelsolin, how G4–G6 binds on the opposite side of the filament.

### G2G3 primes F-actin for binding and severing by G1 and G4–G6

G2G3 binding induces three interconnected structural changes: it introduces a kink in F-actin at the binding site, alters the orientation and conformation of the main interacting subunit A3, and weakens the interaction of this subunit with the one immediately below toward the barbed end (A5). These effects are best analyzed by comparison with the structure of F-actin, which has ideal helical symmetry. This comparison shows that the changes result from G2G3 wedging into the large concavity formed at the A1 H-cleft/A3 D-loop interface, while L1 binds along A3 and helps pull this subunit slightly outward from the filament axis. Stabilization of the interaction between the A3 D-loop and the A1 H-cleft by G2G3 may also create differential tension within the filament, since the D-loop is dynamic in F-actin (27, 30).

**Figure 2:**
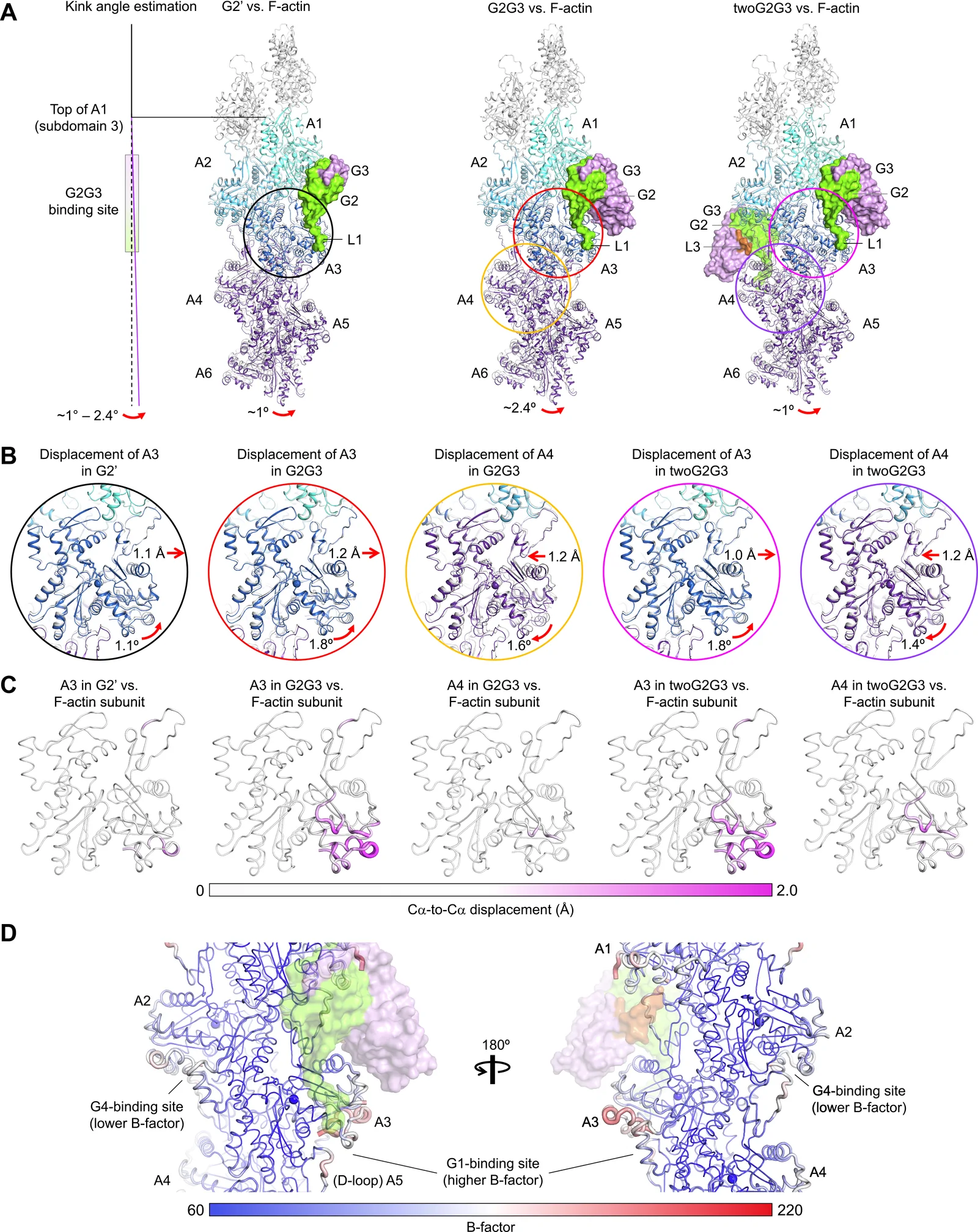
**G2G3-induced conformational changes in F-actin.***A*, Kink in F-actin observed in the G2′, G2G3, and twoG2G3 structures (colored as in Fig. 1) compared to F-actin (PDB 9ZZI, *gray*). A diagram on the left illustrates how the kink is defined and measured. Structural differences in F-actin between the G2′ and G2G3′ states are minimal and thus G2G3′ is not shown. F-actin structures were superimposed based on the inner domain (residues S145–P333) of subunits A1 and A2. *B*, close-up views (corresponding to circles in *A*) showing the displacements and rotations of actin subunits A3 and A4 along the G2G3 binding pathway (Fig. S9). *C*, cartoon putty representation of subunits A3 and A4 colored according to Cα-to-Cα displacement (scale bar shown below) relative to the corresponding F-actin subunits after superposition on the inner domain. *D*, cartoon putty representation of subunits A1–A5 in the G2G3 structure colored by Cα B-factor (scale bar shown below). Residues at the G1 binding site at the A3 H-cleft/A5 D-loop interface exhibit increased mobility, as indicated by higher B-factor values. F-actin, filamentous actin; PDB, Protein Data Bank.

A ∼1° kink in F-actin is already observed in the G2′ structure. It remains essentially unchanged in the G2G3′ structure and increases to ∼2.4° in the fully bound G2G3 structure (Fig. 2*A*). Although small, this kink produces incremental yet substantial displacements of subunits toward the barbed end relative to the G2G3 binding site. Gelsolin-induced bending of F-actin has previously been observed by fluorescence microscopy (31) and low-resolution electron microscopy (32). The G2G3-induced kink observed here likely accounts for this bending and precedes severing, which occurs on binding of the other gelsolin domains (G1 and G4–G6). It is interesting to contrast this behavior with that of cofilin, the other major severing protein, which also induces structural perturbations in the filament, including a change in helical twist and a transition of immediately interacting subunits to a G-actin-like conformation (33), but these changes do not propagate beyond the site of contact (34).

The kink appears to arise from a G2G3-induced rotation of subunit A3 (Fig. 2*B*). This rotation can be described as a tilt of subdomains 1 and 3 away from the filament axis, combined with an anticlockwise azimuthal rotation of A3 (Fig. S9*A*). The pivot point for this rotation is approximately centered on residue V43 at the top of the D-loop, and its magnitude ranges from ∼1.1° in G2′ to ∼1.8° in G2G3. Smaller rotations propagate to subunits beyond the G2G3 binding site, illustrating how the filament buffers the effects of kinking. In addition to rotation, A3 undergoes a translation of ∼1.1 Å in G2′ and ∼1.2 Å in G2G3.

In addition to the overall rotation–translation of A3, direct comparison of this subunit with its counterpart in F-actin, using the inner domain as the reference for superimposition, reveals a small rotation of the outer domain relative to the inner domain (Fig. 2*C*), reflected as a >2° change in the interdomain dihedral angle (Fig. S9*B*). This outer-domain rotation occurs in the same direction, albeit much less pronounced than that observed when G1 is bound to the same subunit in the gelsolin barbed end structure (13).

Together, the rotation–translation of A3 and the small rotation of its outer domain appear to weaken the interaction between A3 and A5. This is supported by increased flexibility at the A3 H-cleft/A5 D-loop interface, as indicated by elevated temperature factors (B-factors) of these residues compared to the equivalent A2 H-cleft/A4 D-loop interface on the opposite side of the filament (Fig. 2*D*). Weakening of contacts between A3 and A5 likely allows G1 to opportunistically bind the H-cleft of A3, as observed in the barbed-end-bound structure (13). By disrupting a key longitudinal contact between subunits along the long-pitch helix, G1 binding represents a critical step in filament severing.

Although the B-factors do not increase at the A2/A4 interface, the kink induced by G2G3 binding on one side of the filament also affects the opposite side. Consequently, A4 undergoes an overall rotation–translation similar in magnitude to that of A3 but in the opposite direction (*i.e.*, toward the filament axis) (Fig. 2*B*). In contrast to A3, however, A4 mostly does not exhibit outer-domain movement relative to the inner domain, linking the outer-domain rotation of A3 specifically to its interaction with G2G3 (Figs. 2*C* and S9*B*). The structural changes at the A2/A4 interface likely underlie the limited cooperativity described above (Fig. 1*H*), enabling binding of a second G2G3 molecule at this site or of G4–G6 in the context of full-length gelsolin. Interestingly, in the twoG2G3 structure, we observe a partial reversal of both the filament kink and the overall rotations of A3 and A4 (Figs. 2, *A*, *B* and S9). Because this reversal is incomplete, the first G2G3 molecule, bound closer to the pointed end, appears to play a dominant role in the interaction; that is, the two G2G3 molecules are not fully equivalent.

### G4 acquires severing activity when primed by G2G3

**Figure 3:**
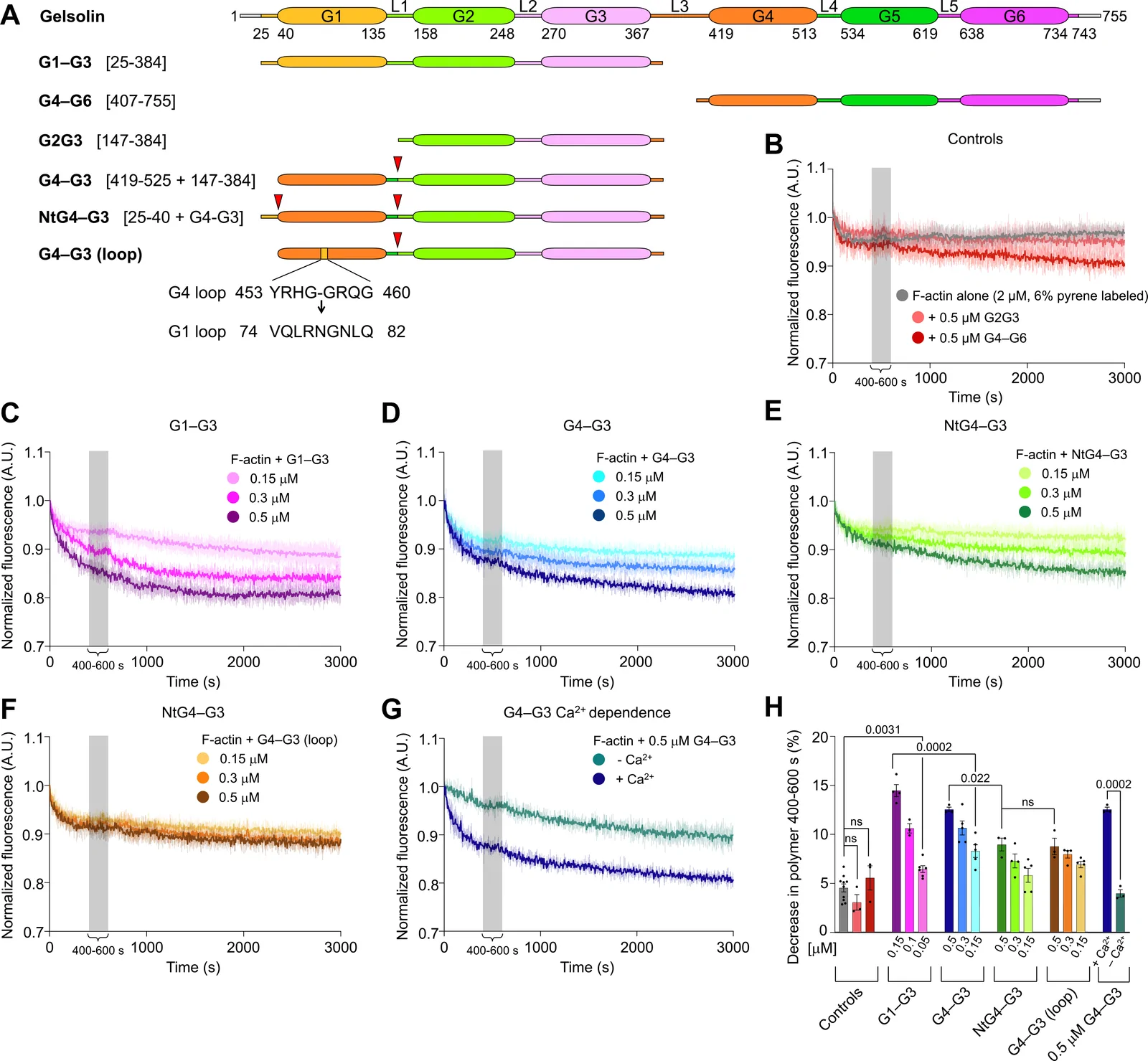
**G4 can partially substitute for G1 within G1–G3.***A*, schematic representation of the gelsolin constructs used in this study (colored as in Fig. 1*A*). *Red arrowheads* indicate the sites of fusion of hybrid constructs. *B*–*G*, time courses of depolymerization of F-actin alone (2 μM, 6% pyrene-labeled) or in the presence of the indicated concentrations of gelsolin constructs. For each condition, *dark traces* represent the mean of three independent experiments with s.d. in *lighter color*. All the experiments, except the -Ca^2+^ condition in panel G, were performed in the presence of 200 μM CaCl_2_. *Gray shading* highlights the 400 to 600 s time interval used to calculate the average decrease in polymer level, reported as the percent change from time = 0. *H*, average decrease in polymer level from time = 0 (data shown as mean ± SEM). Statistical significance was calculated using an unpaired, two-tailed *t* test with Welch's correction, with *p* values reported in the figure and/or Table S1 (ns, *p* > 0.05). F-actin, filamentous actin.

Among the gelsolin domains, G4 is the most closely related to G1, both in sequence and structure (6) (Fig. S10). Both domains engage actin in a similar manner, targeting the H-cleft *via* their long α-helix, and are the only two domains that harbor a type I Ca^2+^-binding site whose coordination involves actin residue E167 (11, 12, 13, 35). Specifically, for human gelsolin studied here, G1 and G4 share 37% sequence identity, with greater conservation on the actin-binding side, including the long helix of the domain (Fig. S10*B*). Despite these similarities, G1 is thought to be the only gelsolin domain capable of severing F-actin, whereas the remaining domains perform auxiliary functions, either mediating F-actin binding or acting synergistically with G1 to enhance the severing activity of full-length gelsolin (6, 19, 20, 21, 22, 23, 25). Thus, recruitment of G1 to F-actin *via* either L1 (19) or the unrelated actin-binding domain of α-actinin (20) is sufficient to elicit measurable severing activity. Having established here that G2G3 primes F-actin for G1-mediated severing, we asked whether G4 could acquire severing activity when combined with the priming activity of G2G3. To address this question, we generated a hybrid construct in which G4 replaces G1 within the N-terminal fragment G1–G3 that accounts for most of the severing activity. The resulting hybrid construct, termed G4–G3, consists of domain G4 (residues Q419–S525) fused N terminally to G2G3 (residues S147–S384). Control constructs included G1–G3, G2G3, and G4–G6 (Fig. 3*A*).

Using the pyrene-actin polymerization assay (36), we analyzed the effect of gelsolin constructs on pre-polymerized F-actin seeds (final concentration: 2 μM, 6% pyrene-labeled) in the presence of 200 μM Ca^2+^ (see below) by monitoring fluorescence changes associated with polymerization and depolymerization. Alone, the actin seeds exhibited a modest yet noticeable decrease in polymer level during the first 100 to 200 s, likely reflecting dilution-driven depolymerization, followed by a slow monotonic increase as the system approached steady-state treadmilling (Fig. 3, *B* and *H*). A similar behavior was observed on addition of 0.5 μM G2G3, consistent with its lack of effect on severing, monomer binding, and filament end capping. Addition of 0.5 μM G4–G6 produced the same initial drop as actin alone, but, unlike actin alone, the signal continued to decline and did not recover, consistent with Ca^2+^-dependent monomer sequestration by G4–G6. In contrast, G1–G3 reduced the polymer level in a concentration-dependent manner, with a significant reduction already evident at 50 nM, consistent with the strong severing activity of this fragment (Fig. 3*C*). These results reproduce previous observations (19, 20, 21, 22, 23, 25) and provide a benchmark for analysis of the G4–G3 hybrid construct.

Like G1–G3, G4–G3 also produced a substantial decrease in the polymer level, although this required approximately 3-fold higher concentrations than G1–G3 (Fig. 3*D*). Importantly, contrary to G1–G3 (7, 22, 23, 24), the activity of G4–G3 showed a strong Ca^2+^-dependence (Fig. 3*G*), which prompted us to perform these experiments in the presence of 200 μM Ca^2+^. Due to the initial drop in polymer level observed with the actin control, we cannot reliably calculate depolymerization rates of the various constructs from the initial phase of the reaction, which would have allowed direct comparisons among the various constructs. We therefore used the average polymer level between 400 and 600 s, late enough to avoid the initial dilution phase but early enough to precede monomer sequestration (Fig. 3*H*). At 0.15 μM, a common concentration used with both G1–G3 and G4–G3, the polymer level dropped by 14.5 ± 0.6% with G1–G3 *versus* 8.3 ± 0.6% with G4–G3 in the presence of Ca^2+^. At 0.5 μM, the lowest concentration at which G4–G3 exhibited detectable activity in the absence of Ca^2+^, the polymer level decreased by 12.5 ± 0.2% *versus* 4.0 ± 0.4% with and without Ca^2+^, respectively (Fig. 3*H*). From these results, we can draw three main conclusions. First, G4, previously thought to lack severing activity, can be potentiated by conformational changes in F-actin induced by G2G3, enabling it to achieve substantial severing activity. Second, the severing activity of G4–G3 is strongly potentiated by Ca^2+^. Third, G1 appears uniquely adapted for efficient severing, with activity that G4 cannot fully recapitulate despite their structural and sequence similarities.

This raises a key question: why is G1 more effective than G4 at severing? Despite the similarities between the two domains described above (Fig. S10, *A*–*C*), subtle differences are observed at the contact interface with actin subunit A3. In particular, G1 residue F49 and L1 residue F149 form a small hydrophobic cluster, which is disrupted in G4, where the equivalent residue S428 may not cooperate with F149 during the initial approach and insertion of the long helix into the H-cleft of A3 (Fig. S10, *D* and *E*). Larger differences affect the surface that faces subunit A5, whose D-loop must be displaced from the H-cleft of A3 for the long helix to insert into this cleft. This region includes the loop ^74^VQLRNGNLQ^82^ of G1, whose sequence differs in G4 and contains a one-amino acid deletion (^453^YRHG-GRQG^460^) (Figs. 3*A* and S10*A*). Such differences could influence how efficiently each domain perturbs the D-loop–H-cleft interaction at the severing interface. A second distinction lies not within the domains themselves, but in the loops preceding them, which differ in sequence and adopt distinct conformations in the structure of full-length gelsolin (13) (^25^MVVEHPEFLKAGKEPG^40^ *versus* ^404^TAMAAQHGMDDDGTGQ^419^) (Fig. S10*A*). Most studies concluding that G1 is essential for severing included this N terminal tail. One study, however, performed incremental N-terminal deletions and found that removal of residues 25 to 35 reduced severing activity, whereas a larger deletion (residues 25–41) completely abolished it. The latter deletion, however, extends into the first β-strand of the domain, which likely compromises folding (24).

To test whether the N-terminal tail and the A5-facing loop contribute to the higher severing activity of G1–G3, we generated two hybrid constructs, one adding the N-terminal tail to G4–G3 (NtG4–G3) and the other replacing the loop 453 to 460 of G4 with G1 residues 74 to 82 within construct G4–G3, termed G4–G3 (loop) (Fig. 3*A*). Contrary to our initial expectation, neither construct enhanced the severing activity of G4–G3; instead, both reduced it (Fig. 3, *E*, *F* and *H*). This may reflect either a more limited contribution of these regions to severing than anticipated or, alternatively and perhaps more likely, incorrect structural positioning of the grafted segments within the hybrid constructs due to altered intramolecular interactions with G4 compared to their native interactions with G1.

In summary, this study sought to determine whether G2G3, previously known as gelsolin’s Ca^2+^-independent F-actin–binding region (18), has additional roles during severing. We find that, in addition to recruiting other gelsolin domains, G2G3 is directly responsible for the previously observed gelsolin-induced filament bending (31, 32) and induces local conformational changes in the protomers at the severing interface that facilitate the severing activity of the other gelsolin domains. Thus, while previous studies had concluded that G1 was indispensable for severing, such that any fragment achieving substantial severing activity had to include G1, we found that the related G4 domain can substitute for G1 to a considerable extent when primed by G2G3, albeit requiring the presence of Ca^2+^. G4’s dependence on Ca^2+^ for severing appears to mirror the Ca^2+^ dependence of G4–G6 for G-actin binding (21, 22, 23). Therefore, G1 still emerges as uniquely effective at severing, which, together with the Ca^2+^-independence of this activity, accounts for the particularly strong severing capacity of the G1–G3 fragment generated during apoptosis (7). To conclude, our results, together with our previous structure of full-length gelsolin at the barbed end (13), allow us to propose an updated model of gelsolin-mediated severing, which is presented as an animation in Movie S1.

## Experimental procedures

### Proteins

Skeletal α-actin was purified from fresh hind leg rabbit muscle tissue purchased from Pel-Freeze Biologicals (37). Constructs G2G3, MBP–G1–G3, MBP–G4–G3, and MBP–G4–G6 were PCR-amplified from a pRSF1 vector (Novagen) containing the coding sequence for full-length human gelsolin (13). Primers used in this study are listed in Table S2.

#### Cloning

G2G3 was generated using primers annealing within linker L1 (starting at S147) and spanning to S384 while introducing a C-terminal Strep tag. The PCR product was cloned between the SapI and EcoRI sites of pTYB11 (NEB). MBP–G1–G3 was generated by amplifying G1–G3 (M25–S384) and introducing a C-terminal Strep tag. MBP–G4–G6 was amplified to encode residues A407 through the C terminus. MBP–G4–G3 was assembled by ligating a G4 fragment (residues Q419–S525) with a G2G3 fragment using a BspEI restriction site, followed by cloning into pMAL-c6T between the BamHI and EcoRI sites. MBP–G4–G3 was modified through three rounds of amplification with the coding sequence for residues M25–G40 incorporated into the forward primer to produce construct NtG4–G3. The MBP–G4–G3 (loop) construct was generated through successive rounds of site-directed mutagenesis using the QuikChange II mutagenesis kit (Agilent Technologies).

#### Protein expression

All constructs were expressed in Arctic Express (DE3) RIL cells (Agilent Technologies) grown in Terrific Broth at 37 °C to an *A*_600_ of 1.2, followed by induction with 0.3 mM isopropyl-β-D-1-thiogalactopyranoside and incubation at 10 °C for 24 h. Cells were harvested by centrifugation at 50,000*g* for 20 min and resuspended in construct-specific buffers as follows: G2G3 in 20 mM Hepes pH 7.5, 500 mM NaCl, 1 mM EDTA; MBP–G1–G3, MBP–G4–G3, and MBP–G4–G6 in 20 mM Hepes pH 7.5, 150 mM NaCl, 1 mM EDTA. All buffers were supplemented with 5 mM benzamidine hydrochloride, 1 mM PMSF, and cOmplete protease inhibitor (Roche). Cells were lysed using a Microfluidizer (Microfluidics).

#### Protein purification

G2G3 was purified using a chitin affinity column followed by intein self-cleavage with 0.5 mM tris(2-carboxyethyl)phosphine (TCEP) at room temperature for 24 h. The protein was further purified using a Strep-Tactin affinity column and concentrated to 4 mg mL^−1^ in 20 mM Hepes pH 7.5, 200 mM NaCl. MBP–G1–G3, MBP–G4–G3, MBP–G4–G6, MBP–NtG4–G3, and MBP–G4–G3 (loop) were purified using amylose and Strep-Tactin (IBA Lifesciences) affinity chromatography and concentrated to about 10 mg mL^−1^ in 20 mM Hepes pH 7.5, 150 mM NaCl. Purified proteins were aliquoted, flash-frozen in liquid nitrogen, and stored at −80 °C.

### Cryo-EM sample preparation, data acquisition, and processing

ATP-actin (57 μM) in G-buffer (2 mM Tris pH 8.0, 0.2 mM CaCl_2_, 0.2 mM ATP, 0.5 mM dithiothreitol (DTT)) was added to a 50 μl solution containing 10 μM G2G3 in F-actin buffer (20 mM Hepes pH 7.5, 50 mM KCl, 0.5 mM CaCl_2_, 0.5 mM MgCl_2_, 0.2 mM ATP, and 0.5 mM DTT), yielding a final actin concentration of 10 μM. The mixture was incubated at room temperature for 2 h. This high G2G3:actin ratio was used to promote maximal decoration of F-actin filaments by G2G3.

For cryo-EM, 3 μl of sample was applied to glow-discharged (90 s, PELCO easiGlow) 200-mesh 1.2/1.3 C-flat 2Cu-50 carbon grids (Electron Microscopy Sciences). Grids were blotted for 2.5 s with a force of 7 using Whatman 41 filter paper and vitrified by plunge-freezing into liquid ethane using a Vitrobot Mark IV (Thermo Fisher Scientific).

Cryo-EM data were collected over three sessions using EPU (Thermo Fisher Scientific) on FEI Titan Krios microscopes operating at 300 kV and equipped with a Gatan K3 direct electron detector with an energy filter. Movies were recorded at a nominal magnification of ×81,000 in super-resolution mode, yielding a pixel size of 0.54 Å. The defocus range was −0.5 to −2.5 μm, and the total electron dose was 45 e^−^/Å^2^.

A total of 13,423 movies were processed in CryoSPARC v4.4 (38). Motion correction was performed with twofold binning, resulting in a pixel size of 1.08 Å. Micrographs with a contrast transfer function (CTF) fit worse than 6 Å were excluded after patch CTF estimation, leaving 12,162 micrographs. Particle picking was performed using a filament tracer, yielding 39,567,874 particles. Particles were extracted using a box size of 320 pixels (346 Å) and binned twofold to 128 pixels. Reference-free 2D classification removed non–F-actin particles. Accepted particles were used for *ab initio* reconstruction of an F-actin map, whereas rejected particles were used to generate a “junk” class. Heterogeneous refinement using these volumes yielded a final F-actin dataset of 4,643,974 particles, which showed weak additional density for G2G3 at eight sites along the filament.

To address misalignment arising from longitudinal particle shifts, focused 3D classification without image alignment was performed using masks centered on each of the eight binding sites. This enabled recentering of particles toward the dominant filament register. Multiple particle classes corresponding to different binding states of G2G3 were identified and separated into three structural states. The undecorated particle stack was processed separately by helical reconstruction to generate a reference F-actin structure at 2.3 Å resolution.

For the twoG2G3-bound state, a second round of focused 3D classification was performed starting from the G2G3-enriched particle stack, using a mask placed on the opposite side of the filament. This yielded additional classes that were processed as above.

Final particle stacks for G2′, G2G3′, G2G3, and twoG2G3 contained 328,926, 183,304, 698,879, and 39,272 particles, respectively. These were subjected to global and local CTF refinement, reference-based motion correction, and nonuniform refinement in CryoSPARC, yielding final consensus maps at resolutions of 2.86 Å, 3.03 Å, 2.76 Å, and 3.44 Å, respectively (Fig. S2).

Map quality was assessed using 3DFSC (39) and cryoEF (40). 3DFSC sphericity values were 0.985, 0.982, 0.975, and 0.982 for G2′, G2G3′, G2G3, and twoG2G3 maps, respectively. Orientation distribution efficiencies (Eod) from cryoEF were 0.76, 0.75, 0.75, and 0.75, respectively. Local resolution estimates were obtained in CryoSPARC using an Fourier Shell Correlation (FSC) cutoff of 0.5 (Table 1 and Figs. S3–S6).

### Model building and refinement

Models were built in Coot (41) and refined in Phenix (42) starting from a high-resolution F-actin structure (PDB 9ZZI) and a gelsolin-barbed-end complex (PDB 8VIZ) (13). All five models were refined using real-space refinement in Phenix with individual atomic positions and B-factors. Figures were prepared in PyMOL (Schrödinger, LLC) and ChimeraX (43).

### Actin polymerization/depolymerization assay

Assays were performed using a Cary Eclipse fluorescence spectrophotometer (Varian). Mg-ATP F-actin seeds (20 μM, 6% pyrene-labeled) were polymerized at room temperature for 30 min, then at 4 °C for 16 h, all in the dark. Gelsolin constructs were added directly to the concentrated seeds, and the mixture was immediately transferred by Pasteur pipet into a cuvette containing Mg^2+^-G-buffer supplemented with 200 μM CaCl_2_, diluting the seeds to a final concentration of 2 μM (Fig. 3). Mixing was performed gently to avoid filament shearing, and recording began approximately 30 s after mixing. Average percent depolymerization in the 400 to 600 s interval was calculated as (1–average normalized fluorescence from 400–600 s) × 100. Statistical analysis was performed in Prism using an unpaired *t* test with Welch’s correction; *p* values are reported in the figures and in Table S1.

### F-actin cosedimentation assay

Rabbit skeletal α-actin (40 μM) was polymerized in ATP-supplemented F-buffer (20 mM HEPES pH 7.5, 50 mM KCl, 1 mM EGTA, 1 mM MgCl₂, 1 mM ATP, and 1 mM DTT). F-actin (6 μM final) was incubated with increasing concentrations of G2G3 (Fig. S8). To ensure Ca^2+^-dependent binding, EGTA was omitted and samples were supplemented with 20 mM CaCl_2_. Reactions (100 μl) were incubated at 4 °C for 1 h and centrifuged at 80,000 rpm for 30 min using a TLA-100 rotor.

Supernatants were mixed with 4× SDS loading buffer. Pellets were washed with KMI buffer (containing Ca^2+^ and ATP), resuspended in fresh buffer, and mixed with loading buffer. Samples were analyzed by SDS-PAGE and Coomassie staining, imaged on a G:BOX system (Syngene), and quantified densitometrically using Image Lab (Bio-Rad). Data were fit in Prism (v11.0.0) using nonlinear regression to a specific binding model with a Hill slope.

### Definition of subunit coordinate frames and angle calculations

Centers of mass (CoMs) were calculated for residues 6 to 374 of each actin subunit. Subdomains were defined as SD1 (7–31, 74–136, 347–364), SD2 (34–39, 53–68), SD3 (149–179, 274–331), and SD4 (185–256), excluding flexible linkers.

The filament axis was defined by principal component analysis of subunit CoMs. Local coordinate frames were constructed as follows: the Z-axis (tilt) connects SD3 to SD4, the Y-axis (azimuthal) is the radial vector from the filament axis to the subunit CoM (after orthogonal projection), and the X-axis (twist) is defined as the cross product of Y and Z.

For each longitudinal pair of subunits, the reference filament was aligned using residues 6 to 375. Rotation matrices between corresponding subunits were computed and converted to axis–angle representations. These were decomposed into tilt, azimuthal, and twist components in the local frame. Values below 0.01° were set to zero. Dihedral angles were computed from SD2−SD1–SD3–SD4 CoMs using a standard four-point torsion definition.

## Ethics statement

This study did not involve experiments with human participants or animals.

## Data availability

Molecular models and cryo-EM density maps have been deposited with the following accession codes: G2’ (PDB ID 12BW, EMD-76298), G2G3’ (PDB ID 12BX, EMD-76299), G2G3 (PDB ID 12BY, EMD-76300), twoG2G3 (PDB ID 12BZ, EMD-76301).

## Supporting information

This article contains supporting information (13, 27, 29, 39, 40, 42, 44).

## Conflict of interest

The authors declare that they have no conflicts of interest with the contents of this article.

## References

[1] Ono S., Mohri K., Ono K. (2004). Microscopic evidence that actin-interacting protein 1 actively disassembles actin-depolymerizing factor/Cofilin-bound actin filaments. J. Biol. Chem..

[2] Chen Q., Courtemanche N., Pollard T.D. (2015). Aip1 promotes actin filament severing by cofilin and regulates constriction of the cytokinetic contractile ring. J. Biol. Chem..

[3] Kotila T., Wioland H., Enkavi G., Kogan K., Vattulainen I., Jegou A. (2019). Mechanism of synergistic actin filament pointed end depolymerization by cyclase-associated protein and cofilin. Nat. Commun..

[4] Shekhar S., Chung J., Kondev J., Gelles J., Goode B.L. (2019). Synergy between Cyclase-associated protein and Cofilin accelerates actin filament depolymerization by two orders of magnitude. Nat. Commun..

[5] Yin H.L., Stossel T.P. (1979). Control of cytoplasmic actin gel-sol transformation by gelsolin, a calcium-dependent regulatory protein. Nature.

[6] Nag S., Larsson M., Robinson R.C., Burtnick L.D. (2013). Gelsolin: the tail of a molecular gymnast. Cytoskeleton.

[7] Kothakota S., Azuma T., Reinhard C., Klippel A., Tang J., Chu K. (1997). Caspase-3-generated fragment of gelsolin: effector of morphological change in apoptosis. Science.

[8] Kwiatkowski D.J. (1999). Functions of gelsolin: motility, signaling, apoptosis, cancer. Curr. Opin. Cell Biol..

[9] Lee W.M., Galbraith R.M. (1992). The extracellular actin-scavenger system and actin toxicity. N. Engl. J. Med..

[10] Burtnick L.D., Koepf E.K., Grimes J., Jones E.Y., Stuart D.I., McLaughlin P.J. (1997). The crystal structure of plasma gelsolin: implications for actin severing, capping, and nucleation. Cell.

[11] Robinson R.C., Mejillano M., Le V.P., Burtnick L.D., Yin H.L., Choe S. (1999). Domain movement in gelsolin: a calcium-activated switch. Science.

[12] Burtnick L.D., Urosev D., Irobi E., Narayan K., Robinson R.C. (2004). Structure of the N-terminal half of gelsolin bound to actin: roles in severing, apoptosis and FAF. Embo J..

[13] Barrie K.R., Rebowski G., Dominguez R. (2025). Mechanism of actin filament severing and capping by gelsolin. Nat. Struct. Mol. Biol..

[14] Choe H., Burtnick L.D., Mejillano M., Yin H.L., Robinson R.C., Choe S. (2002). The calcium activation of gelsolin: insights from the 3A structure of the G4-G6/actin complex. J. Mol. Biol..

[15] Nag S., Ma Q., Wang H., Chumnarnsilpa S., Lee W.L., Larsson M. (2009). Ca2+ binding by domain 2 plays a critical role in the activation and stabilization of gelsolin. Proc. Natl. Acad. Sci. U. S. A..

[16] Janmey P.A., Chaponnier C., Lind S.E., Zaner K.S., Stossel T.P., Yin H.L. (1985). Interactions of gelsolin and gelsolin-actin complexes with actin. Effects of calcium on actin nucleation, filament severing, and end blocking. Biochemistry.

[17] Yin H.L., Iida K., Janmey P.A. (1988). Identification of a polyphosphoinositide-modulated domain in gelsolin which binds to the sides of actin filaments. J. Cell Biol..

[18] Bryan J. (1988). Gelsolin has three actin-binding sites. J. Cell Biol..

[19] Kwiatkowski D.J., Janmey P.A., Yin H.L. (1989). Identification of critical functional and regulatory domains in gelsolin. J. Cell Biol..

[20] Way M., Pope B., Weeds A.G. (1992). Evidence for functional homology in the F-actin binding domains of gelsolin and alpha-actinin: implications for the requirements of severing and capping. J. Cell Biol..

[21] Bryan J., Hwo S. (1986). Definition of an N-terminal actin-binding domain and a C-terminal Ca2+ regulatory domain in human brevin. J. Cell Biol..

[22] Chaponnier C., Janmey P.A., Yin H.L. (1986). The actin filament-severing domain of plasma gelsolin. J. Cell Biol..

[23] Way M., Gooch J., Pope B., Weeds A.G. (1989). Expression of human plasma gelsolin in Escherichia coli and dissection of actin binding sites by segmental deletion mutagenesis. J. Cell Biol..

[24] Peddada N., Sagar A., Rathore Y.S., Choudhary V., Pattnaik U.B., Khatri N. (2013). Global shapes of F-actin depolymerization-competent minimal gelsolins: insight into the role of g2-g3 linker in pH/Ca2+ insensitivity of the first half. J. Biol. Chem..

[25] Selden L.A., Kinosian H.J., Newman J., Lincoln B., Hurwitz C., Gershman L.C. (1998). Severing of F-actin by the amino-terminal half of gelsolin suggests internal cooperativity in gelsolin. Biophys. J..

[26] Pope B., Way M., Weeds A.G. (1991). Two of the three actin-binding domains of gelsolin bind to the same subdomain of actin. Implications of capping and severing mechanisms. FEBS Lett..

[27] Robinson R.C., Chongrungreang T., Ponlachantra K., Boonyarit B., Dilly G.F., Li Y.I. (2025). The structure of an actin nucleus stabilized by villin. Sci. Adv..

[28] Carman P.J., Barrie K.R., Rebowski G., Dominguez R. (2023). Structures of the free and capped ends of the actin filament. Science.

[29] Oosterheert W., Klink B.U., Belyy A., Pospich S., Raunser S. (2022). Structural basis of actin filament assembly and aging. Nature.

[30] Brotzman S.B., Palmer N.J., Boczkowska M., Dominguez R. (2026). Mechanism of actin thin filament pointed-end elongation by leiomodin. Nat. Commun..

[31] Bearer E.L. (1991). Direct observation of actin filament severing by gelsolin and binding by gCap39 and CapZ. J. Cell Biol..

[32] McGough A., Chiu W., Way M. (1998). Determination of the gelsolin binding site on F-actin: implications for severing and capping. Biophys. J..

[33] Palmer N.J., Boczkowska M., Rebowski G., Dominguez R. (2026). Mechanisms of disassembly at the actin filament pointed and barbed ends. Sci. Adv..

[34] Oosterheert W., Boiero Sanders M., Hofnagel O., Bieling P., Raunser S. (2025). Choreography of rapid actin filament disassembly by coronin, cofilin, and AIP1. Cell.

[35] McLaughlin P.J., Gooch J.T., Mannherz H.G., Weeds A.G. (1993). Structure of gelsolin segment 1-actin complex and the mechanism of filament severing. Nature.

[36] Doolittle L.K., Rosen M.K., Padrick S.B. (2013). Measurement and analysis of in vitro actin polymerization. Methods Mol. Biol..

[37] Pardee J.D., Spudich J.A. (1982). Purification of muscle actin. Methods Enzymol..

[38] Punjani A., Rubinstein J.L., Fleet D.J., Brubaker M.A. (2017). cryoSPARC: algorithms for rapid unsupervised cryo-EM structure determination. Nat. Methods.

[39] Tan Y.Z., Baldwin P.R., Davis J.H., Williamson J.R., Potter C.S., Carragher B. (2017). Addressing preferred specimen orientation in single-particle cryo-EM through tilting. Nat. Methods.

[40] Naydenova K., Russo C.J. (2017). Measuring the effects of particle orientation to improve the efficiency of electron cryomicroscopy. Nat. Commun..

[41] Casanal A., Lohkamp B., Emsley P. (2020). Current developments in Coot for macromolecular model building of Electron Cryo-microscopy and Crystallographic Data. Protein Sci..

[42] van Zundert G.C.P., Moriarty N.W., Sobolev O.V., Adams P.D., Borrelli K.W. (2021). Macromolecular refinement of X-ray and cryoelectron microscopy structures with Phenix/OPLS3e for improved structure and ligand quality. Structure.

[43] Pettersen E.F., Goddard T.D., Huang C.C., Meng E.C., Couch G.S., Croll T.I. (2021). UCSF ChimeraX: structure visualization for researchers, educators, and developers. Protein Sci..

[44] Procter J.B., Carstairs G.M., Soares B., Mourao K., Ofoegbu T.C., Barton D. (2021). Alignment of Biological Sequences with Jalview. Methods Mol. Biol..

