# Supplementary material for: Gelsolin domains G2G3 recruit other domains and prime the actin filament for severing
Source: J Biol Chem. 2026 Aug 7;302(9):113413. doi: 10.1016/j.jbc.2026.113413 (PMC13553588; doi:10.1016/j.jbc.2026.113413)
Supplement: Supplementary file 1 [file mmc1.docx]

Supporting Information for

Gelsolin domains G2G3 recruit other domains and prime the actin filament for severing

Andrew J. Saks, Malgorzata Boczkowska, Kyle R. Barrie, Robert C. Robinson, Roberto Dominguez

**This PDF file includes:**

Figures S1 to S10

Table S1 to S2

Legend for Movie S1

SI References

**Figure S1. Structure of full-length gelsolin at the barbed end of F-actin.** (A) Domain diagram of human gelsolin, including the 24–amino acid N-terminal extension of plasma gelsolin. (B) Structure of full-length gelsolin at the barbed end of the actin filament (PDB: 8VIZ) (1). Gelsolin domains are colored according to the diagram shown in part A, and actin subunits are colored according to Fig. 1 in the main text. Eight Ca^2+^ ions are shown (red spheres), including six type-II sites bound one per gelsolin domain and two type-I sites located at the interfaces of G1 and G4 with F-actin.

Figure S2. Cryo-EM data processing workflow. (A) Representative micrograph. (B) Representative 2D class averages. White arrows indicate extra density corresponding to G2G3. (C) Data processing workflow. The final maps used in model building and refinement are colored and labeled.

**Figure** **S3. Map quality and resolution assessment (G2’ structure).** (A) Half-map Fourier shell correlation (FSC) resolution analysis of the consensus map that led to the two combined maps used in refinements (2.86 Å at FSC = 0.143). (B) Orientation distribution of particles used in the consensus map determined with the program cryoEF (2). The consensus map has a calculated efficiency (E_od_) of 0.76. (C) 3D Fourier shell correlation calculated using 3DFSC (3). The consensus map has a sphericity of 0.985 and a global resolution of 2.98 Å. (D) Map to model FSC determined with the program Phenix (4). (E) Cryo-EM maps colored by local resolution (scale bar to the right).

Figure S4. Map quality and resolution assessment (G2G3’ structure). (A) Half-map Fourier shell correlation (FSC) resolution analysis of the consensus map that led to the two combined maps used in refinements (3.03 Å at FSC = 0.143). (B) Orientation distribution of particles used in the consensus map determined with the program cryoEF (2). The consensus map has a calculated efficiency (E_od_) of 0.75. (C) 3D Fourier shell correlation calculated using 3DFSC (3). The consensus map has a sphericity of 0.982 and a global resolution of 3.14 Å. (D) Map to model FSC determined with the program Phenix (4). (E) Cryo-EM maps colored by local resolution (scale bar to the right).

Figure S5. Map quality and resolution assessment (G2G3 structure). (A) Half-map Fourier shell correlation (FSC) resolution analysis of the consensus map that led to the two combined maps used in refinements (2.76 Å at FSC = 0.143). (B) Orientation distribution of particles used in the consensus map determined with the program cryoEF (2). The consensus map has a calculated efficiency (E_od_) of 0.75. (C) 3D Fourier shell correlation calculated using 3DFSC (3). The consensus map has a sphericity of 0.975 and a global resolution of 2.79 Å. (D) Map to model FSC determined with the program Phenix (4). (E) Cryo-EM maps colored by local resolution (scale bar to the right).

Figure S6. Map quality and resolution assessment (twoG2G3 structure). (A) Half-map Fourier shell correlation (FSC) resolution analysis of the consensus map that led to the two combined maps used in refinements (3.44 Å at FSC = 0.143). (B) Orientation distribution of particles used in the consensus map determined with the program cryoEF (2). The consensus map has a calculated efficiency (E_od_) of 0.75. (C) 3D Fourier shell correlation calculated using 3DFSC (3). The consensus map has a sphericity of 0.982 and a global resolution of 3.72 Å. (D) Map to model FSC determined with the program Phenix (4). (E) Cryo-EM maps colored by local resolution (scale bar to the right).

**Figure S7. Identity of the bound cation.** (A) Nucleotide-binding cleft in the reference F-actin structure determined by helical reconstruction and fitted to the 2.3-Å resolution cryo-EM map. (B, C) Superimpositions of our reference F-actin structure to the previously reported 2.2-Å structures of Mg^2+^-ADP (left) and Ca^2+^-ADP (right) F-actin (5). Red arrows in panel C indicate differences in the conformation of the nucleotide, position of the cation, and conformation of the S14 loop (D11–L18) between our structure and that of Ca^2+^-ADP-actin. These elements superimpose well with the Mg^2+^-ADP-actin structure, indicating our structures also contain Mg^2+^ bound.

**Figure S8. SDS-PAGE analysis of G2G3 cosedimentation assays with F-actin.** These gels were used for quantification of G2G3 cosedimentation with F-actin (as shown in Fig. 1H).

**Figure S9. Movements of actin subunits upon G2G3 binding.** (A) Heatmaps showing the magnitude of rotational differences of subunits relative to F-actin. The heatmaps were generated by aligning the inner domain of each subunit (residues 150–330; subdomains SD3–SD4) to the corresponding reference F-actin subunit. A triangle was then defined between two longitudinally related subunits: the base of the short edge lies on the measured subunit, whereas the long edge carries a marked point corresponding to the reference subunit. The angular displacement of the measured subunit relative to F-actin was calculated from this geometry. (B) Interdomain dihedral angles for each subunit, defined as shown in the diagram on the left, using the centers of mass of the four actin subdomains (Experimental procedures) (6). Typical values are approximately −24° for G-actin and −6.2° for F-actin.

**Figure S10. Comparison of the sequences and structures of gelsolin domains G1 and G4.** (A) Sequence alignments of domains G1 and G4 from different species (performed separately for each domain). Jalview (7) conservation scores for the combined domains are shown at the bottom. Background coloring indicates sequence conservation within each domain: dark blue, mid-blue, light-blue, and white correspond to 100%, >80%, 30–80%, and <30% conservation, respectively. Secondary structure elements are indicated by the diagram above the sequences, where rectangles and arrows denote α-helices and β-strands, respectively. Residues contoured in magenta fall near the H-cleft of A3 (or A2), whereas those contoured in cyan are expected to participate in displacing the D-loop of A5 (or A4) during G1 (or G4) binding. (B) Structure of G1, shown as a ribbon with a semi-transparent surface colored according to per-residue conservation scores across G1 and G4 (from part A; scale bar at the bottom). Residues that contact the H-cleft of A3 (or A2) tend to be more highly conserved (green surface), whereas the opposite is true for residues that face A5 (or A4). (C) Structural superposition of G1, L1, and G4, colored as in Fig. 1A. (D) Perpendicular views of a structural superimposition of G1 and G4 on F-actin, showing side chains for residues within 4 Å of A3. (E) Close-up of L1 residue F149 (green) and surrounding residues in G1 (yellow) (or G4, orange).

Table S1. P-values for pairwise comparisons shown in Figure 3G

| G4–G3 (loop) | 0.15 |  |  |  |  |  |  |  |  |  |  |  |  |  |  | – |
| --- | --- | --- | --- | --- | --- | --- | --- | --- | --- | --- | --- | --- | --- | --- | --- | --- |
|  | 0.3 |  |  |  |  |  |  |  |  |  |  |  |  |  | – | ns |
|  | 0.5 |  |  |  |  |  |  |  |  |  |  |  |  | – | ns | ns |
| NtG4–G3 | 0.15 |  |  |  |  |  |  |  |  |  |  |  | – | 0.0465 | 0.0376 | ns |
|  | 0.3 |  |  |  |  |  |  |  |  |  |  | – | ns | ns | ns | ns |
|  | 0.5 |  |  |  |  |  |  |  |  |  | – | ns | 0.0197 | ns | ns | ns |
| G4–G3 | 0.15 |  |  |  |  |  |  |  |  | – | ns | ns | 0.0335 | ns | ns | ns |
|  | 0.3 |  |  |  |  |  |  |  | – | 0.0396 | ns | 0.0126 | 0.0013 | ns | 0.0159 | 0.0037 |
|  | 0.5 |  |  |  |  |  |  | – | ns | 0.0017 | 0.0220 | 0.0031 | 0.0003 | 0.0375 | 0.0002 | <0.0001 |
| G1–G3 | 0.05 |  |  |  |  |  | – | <0.0001 | 0.0020 | 0.0452 | 0.0402 | ns | ns | ns | 0.0216 | ns |
|  | 0.1 |  |  |  |  | – | 0.0018 | 0.0374 | ns | 0.0277 | ns | 0.0123 | 0.0014 | ns | 0.0109 | 0.0034 |
|  | 0.15 |  |  |  | – | 0.0043 | 0.0001 | 0.0454 | 0.0048 | 0.0002 | 0.0022 | 0.0003 | <0.0001 | 0.0055 | 0.0003 | 0.0002 |
| Controls | G4–G6 |  |  | – | 0.0074 | 0.0403 | ns | 0.026 | 0.0304 | ns | ns | ns | ns | ns | ns | ns |
|  | G2G3 |  | – | ns | 0.0003 | 0.0028 | 0.0335 | 0.0041 | 0.0009 | 0.0049 | 0.0051 | 0.0128 | 0.0491 | 0.0078 | 0.0128 | 0.0256 |
|  | F-actin | – | ns | ns | <0.0001 | 0.0001 | 0.0031 | <0.0001 | 0.0002 | 0.0015 | 0.0058 | 0.0190 | ns | 0.0195 | 0.0001 | 0.0006 |
|  | [µM] |  | 0.5 | 0.5 | 0.15 | 0.1 | 0.05 | 0.5 | 0.3 | 0.15 | 0.5 | 0.3 | 0.15 | 0.5 | 0.3 | 0.15 |
|  |  | F-actin G2G3 G4–G6 | | | G1–G3 | | | G4–G3 | | | NtG4–G3 | | | G4–G3 (loop) | | |

Table S2. Primers used in this study

| Construct | Vector | Forward (starting from 5’) | Reverse (starting from 5’) |
| --- | --- | --- | --- |
| G2G3 | pTYB11 | ggtggttgctcttccaactcaggattcaagcacgtggtaccc | ttcgaattctcacttttcgaactgtggatgactccaggaaaggtaggacaagcccaggc |
| MBP–G1–G3 | pMAL-c6T | ttcggatccatggtggtggaacaccccga | ttcgaattctcacttttcgaactgtggatgactccaggaaaggtaggacaagcccaggc |
| MBP–G4–G6 | pMAL-c6T | ttcctcgaggccgcccagcacgg | ttcaagctttcaggcagccagctcagc |
| MBP–G4–G3 | pMAL-c6T | ttcggatcccagaaacagatctggagaatcgaaggttcc | ttctccggaggaggtgccgcccttgtaga |
|  |  | ttctccggattcaagcacgtggtaccc | ttcgaattctcacttttcgaactgtggatgactccaggaaaggtaggacaagcccaggc |
| MBP–NtG4–G3 | pMAL-c6T | gggaaggagcctggcaaacagatctggagaatcgaaggttccaac | tcacttttcgaactgtggatgactcca |
|  |  | caccccgagttcctcaaggcagggaaggagcctggcaaacag | ttcttcgaattctcacttttcgaactgtggatgactcca |
|  |  | ttcttcggatccatggtggtggaacaccccgagttcctcaaggca |  |
| MBP–G4–G3 (loop) | pMAL-c6T | ggcgacagctacatcattctgtacaacgtccgccatggtggccgc | gcggccaccatggcggacgttgtacagaatgatgtagctgtcgcc |
|  |  | ctacatcattctgtacaacgtccagcatggtggccgcc | ggcggccaccatgctggacgttgtacagaatgatgtag |
|  |  | cattctgtacaacgtccagcttcgtggccgccagggg | cccctggcggccacgaagctggacgttgtacagaatg |
|  |  | caacgtccagcttcgtaaccgccaggggcagata | tatctgcccctggcggttacgaagctggacgttg |
|  |  | aacgtccagcttcgtaacggccaggggcagataatctataactg | cagttatagattatctgcccctggccgttacgaagctggacgtt |
|  |  | ccagcttcgtaacggcaatgggcagataatctataactggc | gccagttatagattatctgcccattgccgttacgaagctgg |
|  |  | gcttcgtaacggcaatctcgggcagataatctataactgg | ccagttatagattatctgcccgagattgccgttacgaagc |
|  |  | gcttcgtaacggcaatctccagcagataatctataactggcagg | cctgccagttatagattatctgctggagattgccgttacgaagc |

**Movie S1 Legend. Animation of structural states, conformational changes in F-actin, and proposed severing model.** The movie begins by illustrating the stepwise mechanism of G2G3 binding to F-actin, integrating cryo-EM maps, atomic models, and conformational transitions between states. Initially, G2 binds (15 s). This step may be particularly relevant for G1–G3 severing under low-calcium conditions, where the calcium-stabilized G2G3 conformation is not achievable in the absence of calcium.

In contrast, at high calcium concentrations G2G3 may adopt its stabilized conformation prior to filament binding. In this case, the interaction may proceed directly to the second stage, with the docking of G2G3 onto the filament (35 s), bypassing the G2-only intermediate, followed by rotation into the fully bound state (45 s). The movie then shows conformational changes in F-actin induced by G2G3 binding, including rotation and translation of subunit A3 (1 min 15 s), accompanied by local filament distortion and kinking.

Finally, the movie integrates these data with our previous structure of full-length Ca^2+^-gelsolin bound at the barbed end (1) to produce an updated severing model (1 min 45 s). In this model, Ca^2+^-activated gelsolin (stabilized by six type-II Ca^2+^ ions bound, one per domain) adopts an open conformation that enables filament binding. G2G3 engages the filament first, bringing along G1 and G4–G6 and inducing local filament kinking.

Subsequently, G1 binds to the H-cleft of subunit A3 in a Ca^2+^-dependent manner (involving an additional type-I Ca^2+^ ion), accompanied by further conformational changes in F-actin. This interaction competes with the A5 D-loop displacing it from the A3 cleft. Although depicted as separate steps in the movie for clarity, these events are likely coordinated and occur simultaneously. Concomitantly, G4–G6 engages the opposite side of the filament. In particular, G4, together with an additional type-I Ca^2+^ ion, binds the H-cleft of subunit A2, competing with and displacing the A4 D-loop. These coordinated interactions and conformational changes destabilize the filament and ultimately lead to severing. Following severing, Ca^2+^-bound gelsolin remains bound to the barbed end, thereby capping the filament.

SI References

1.	Barrie, K. R., Rebowski, G., and Dominguez, R. (2025) Mechanism of actin filament severing and capping by gelsolin *Nat Struct Mol Biol* **32**, 237–242

2.	Naydenova, K., and Russo, C. J. (2017) Measuring the effects of particle orientation to improve the efficiency of electron cryomicroscopy *Nat Commun* **8**, 629

3.	Tan, Y. Z., Baldwin, P. R., Davis, J. H., Williamson, J. R., Potter, C. S., Carragher, B. *et al.* (2017) Addressing preferred specimen orientation in single-particle cryo-EM through tilting *Nat Methods* **14**, 793–796

4.	van Zundert, G. C. P., Moriarty, N. W., Sobolev, O. V., Adams, P. D., and Borrelli, K. W. (2021) Macromolecular refinement of X-ray and cryoelectron microscopy structures with Phenix/OPLS3e for improved structure and ligand quality *Structure* **29**, 913–921 e914

5.	Oosterheert, W., Klink, B. U., Belyy, A., Pospich, S., and Raunser, S. (2022) Structural basis of actin filament assembly and aging *Nature* **611**, 374–379

6.	Robinson, R. C., Chongrungreang, T., Ponlachantra, K., Boonyarit, B., Dilly, G. F., Li, Y. I. *et al.* (2025) The structure of an actin nucleus stabilized by villin *Sci Adv* **11**, eadw6915

7.	Procter, J. B., Carstairs, G. M., Soares, B., Mourao, K., Ofoegbu, T. C., Barton, D. *et al.* (2021) Alignment of Biological Sequences with Jalview *Methods Mol Biol* **2231**, 203–224
